# Dynamic Functional Brain Connectivity for Face Perception

**DOI:** 10.3389/fnhum.2015.00662

**Published:** 2015-12-08

**Authors:** Yuan Yang, Yihong Qiu, Alfred C. Schouten

**Affiliations:** ^1^Department of Biomechanical Engineering, Delft University of TechnologyDelft, Netherlands; ^2^School of Biomedical Engineering, Shanghai Jiao Tong UniversityShanghai, China; ^3^MIRA Institute for Biomedical Technology and Technical Medicine, University of TwenteEnschede, Netherlands

**Keywords:** face perception, dynamic functional connectivity, high-density EEG, phase lag index, ERP

## Abstract

Face perception is mediated by a distributed brain network comprised of the core system at occipito-temporal areas and the extended system at other relevant brain areas involving bilateral hemispheres. In this study we explored how the brain connectivity changes over the time for face-sensitive processing. We investigated the dynamic functional connectivity in face perception by analyzing time-dependent EEG phase synchronization in four different frequency bands: theta (4–7 Hz), alpha (8–14 Hz), beta (15–24 Hz), and gamma (25–45 Hz) bands in the early stages of face processing from 30 to 300 ms. High-density EEG were recorded from subjects who were passively viewing faces, buildings, and chairs. The dynamic connectivity within the core system and between the extended system were investigated. Significant differences between faces and non-faces mainly appear in theta band connectivity: (1) at the time segment of 90–120 ms between parietal area and occipito-temporal area in the right hemisphere, and (2) at the time segment of 150–180 ms between bilateral occipito-temporal areas. These results indicate (1) the importance of theta-band connectivity in the face-sensitive processing, and (2) that different parts of network are involved for the initial stage of face categorization and the stage of face structural encoding.

## Introduction

When looking around, humans can spot faces almost instantaneously. Face perception is one of the highly developed visual recognition skills in primates and human beings. Despite the quick and effortless recognition of faces, face perception involves complex neuronal mechanisms for face-sensitive processing (Ellis, 1986; Rolls et al., 1992; Bentin and Deouell, 2000). Evidence for face-sensitive processing has been shown in many studies (Puce et al., 1995; Nelson, 2001; Halit et al., 2003). Measuring dynamic brain responses while briefly presenting the stimulus (an image of a face), i.e., event-related potentials (ERPs), have been widely used to investigate face perception (Bentin and Deouell, 2000). The excellent temporal resolution of ERPs allows to precisely determine the time courses of face processing. The most well-known face-sensitive ERP component is N170, a negative potential measured over the bilateral occipito-temporal areas at around 170 ms after stimulus onset. Typically, face stimuli will evoke N170 with larger amplitude and shorter latency than non-face stimuli (Jeffreys, 1983, 1989; Jeffreys and Tukmachi, 1992). According to the model proposed by Bruce and Young (1986), N170 is considered to be associated with a key stage of face processing, namely the ‘structural encoding’ (Sagiv and Bentin, 2001). Besides N170, many studies have also found a positive potential sensitive to face stimuli occurring between 100 and 150 ms (P1) after stimulus onset at the bilateral occipito-temporal areas (Linkenkaer-Hansen et al., 1998; Itier and Taylor, 2004b; Herrmann et al., 2005; Latinus and Taylor, 2006; Kuefner et al., 2009). Some researchers suggested that P1 might reflect a preliminary processing stage prior to the face structural encoding (Herrmann et al., 2005; Latinus and Taylor, 2006). N170 is typically followed by a later component P2, which is a positive potential within the same region (Caharel et al., 2002; Itier and Taylor, 2002; Latinus and Taylor, 2006). Together, these three components (P1, N170, and P2) compose an ERP complex, which may reflect the sequential neural activities of face processing at the bilateral occipito-temporal areas (Latinus and Taylor, 2006; Yang et al., 2011; Guo et al., 2013).

Face perception is mediated by a distributed neural network that is comprised of multiple brain regions including the occipito-temporal areas (Haxby et al., 2000). The occipito-temporal areas contain several face-responsive regions, such as fusiform gyrus (FG), inferior occipital gyrus (IOG), and superior temporal sulcus (STS), which play an important role in face perception (Rossion et al., 2003; Schiltz and Rossion, 2006). A few clinical studies found that some brain lesions outside the occipito-temporal areas also affect face perception (Marinkovic et al., 2000; Vuilleumier et al., 2004; Steeves et al., 2006). Single-cell recording in monkeys revealed that face-selective cells exist not only in visual cortex (Desimone, 1991) but also in other non-visual cortices, such as amygdala (Leonard et al., 1985) and prefrontal cortex (Scalaidhe et al., 1997; Tsao et al., 2008). Functional brain imaging studies (e.g., fMRI, PET) with healthy subjects demonstrated that multiple brain regions are involved to process the information from faces to access knowledge of their viewing condition and facial configuration (Sergent et al., 1992; Haxby et al., 1994; Ishai et al., 2005; Fairhall and Ishai, 2007). According to the model proposed by Haxby et al. (2000), the face processing system is divided into two subsystems: a core system and an extended system. The core system, comprised of FG, IOG, and STS in the visual cortex, mainly contributes to the face encoding, where the extended system involves several non-visual cortices, which are recruited to cooperate with the face-responsive regions in the core system for facilitating face processing and extracting the additional meaning from the face stimulus (Haxby et al., 2002).

The topography and latency of face-sensitive ERP reflect the functional mapping and time courses of face processing, in particular for the core system. Single-cell recording and fMRI studies revealed the organization of distributed neural system for face perception. To the best of our knowledge, it has never been explored how the different brain areas dynamically communicate with each other on a milliseconds scale during face perception. To fill this gap, this paper investigates the dynamic functional connectivity between brain areas in early stages (30–330 ms) of face processing. This work focuses on the brain connectivity passively viewing normal upright faces, since these are the most common face stimuli during daily life.

Dynamic functional connectivity can be investigated using either functional imaging (Rajna et al., 2015; Thompson and Fransson, 2015) or neurophysiological techniques (Sakkalis, 2011). Functional imaging approaches such as fMRI have a low temporal resolution due to hemodynamics of the BOLD signal; as a result, fMRI is unable to capture the fast (transient) dynamics in the neural communication of face perception. Although neurophysiological measurements such as EEG and MEG have a high temporal resolution, traditional functional connectivity methods such as coherence and cross-correlation usually have a high risk of false positive when applied to neurophysiological data (Nolte et al., 2004; Stam et al., 2007). Due to the volume conduction effect, recorded EEG/MEG from nearby channels are very likely to pick up similar neural activities from the common sources, which can lead to spurious correlation between these channels. Another unique problem for EEG is that an active reference electrode will contribute similar components to different electrodes and therefore disturbs the estimations of functional connectivity. To overcome these problems, Stam et al. (2007) introduced an alternative measure, namely phase lag index (PLI), to estimate functional connectivity. PLI is based on the idea that volume conduction from a single active source can never generate a consistent, non-zero phase lag (time delay) between two signals; thus, the true connectivity can be quantified by checking the distribution of the phase lag.

In this paper, we used PLI to estimate dynamical functional connectivity in face perception. The EEG data were recorded from healthy subjects when they were passively viewing face and non-face stimuli. The connectivity between the bilateral occipito-temporal areas was analyzed to investigate the inter-hemisphere communication in the core system for face processing. Furthermore, we also examined the connectivity between the occipito-temporal areas and other brain areas to explore the dynamical interaction between the core system and the extended system in face perception.

## Materials and Methods

### Subjects

Ten healthy right-handed volunteers (age: 18–25, five women) with normal or corrected-to-normal vision participated in the experiment. All subjects were naïve to the experiment and without any neurological disorders. They gave written informed consent before the experiment and received a financial reimbursement for participation. The study complied with the Declaration of Helsinki and was conducted in Shanghai Jiao Tong University. The experimental protocol was approved by the Ethics Committee of the School of Biomedical Engineering, Shanghai Jiao Tong University.

### Experimental Protocol

**FIGURE 1:**
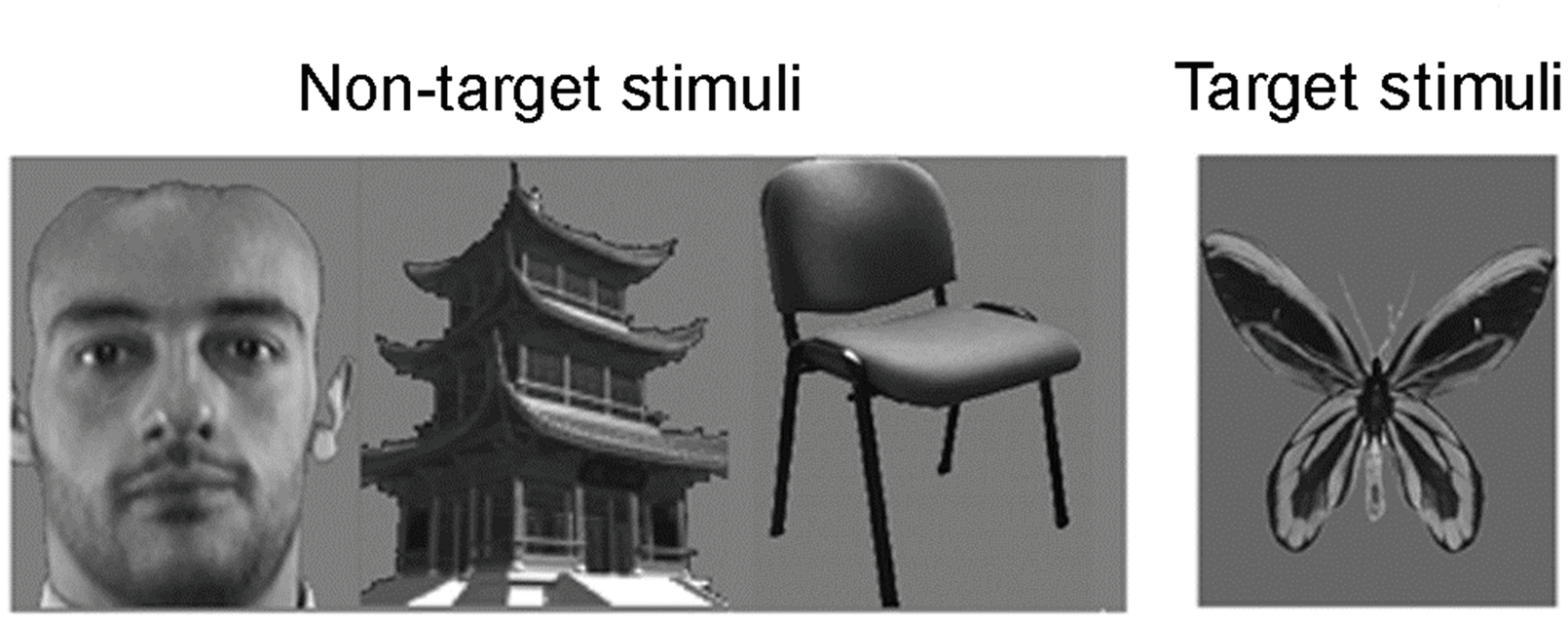
**Examples of the visual stimuli.** Three categories of photographs (224 pixels × 189 pixels) including 10 human faces (5 women), 10 buildings, 10 chairs were used for visual perception. The face photographs were taken from “the database of faces” from AT&T Laboratories Cambridge (Samaria and Harter, 1994) and the facial hair was removed. Additionally, three photographs of butterflies were introduced as target stimuli to help subjects concentrate on the experiment and to eliminate the effect of selective attention on the stimuli. The background of all stimuli was tuned to an 8-bit uniform gray level to uniform the luminance.

The experiments were conducted in a dark sound-radio-frequency shielding room (produced by Union-Brother Soundproof, China). Three categories of photographs (224 pixels × 189 pixels) including 10 human faces (5 women), 10 buildings and 10 chairs were used in this study for comparing the responses to face and non-face stimuli. The face photographs were taken from “the database of faces” from AT&T Laboratories Cambridge (Samaria and Harter, 1994) and the facial hair was removed. Additionally, three photographs of butterflies were used as target stimuli to help subjects concentrate on the experiment. The background of all stimuli was tuned to an 8-bit uniform gray level to uniform the luminance (see **Figure 1**).

Each type of stimuli were presented 12 times with other types of stimulus randomly shown in between. Since all stimuli have the same number of repetitions, that should not affect the comparison between faces and non-faces. Although repeating stimuli may result in the transient working memory effect (old-new effect), this effect will be largely reduced when there are some intervening pictures between repetitions (Itier and Taylor, 2004a). By repeating the stimuli, there were 120 trials per category for faces, buildings and chairs each, and 36 trials for target stimuli (butterflies); giving 396 trials in total. The whole experiment was divided into two blocks; each block consisted of 198 trials and a 2 min break in between. The first block contained 17 target trials, while the second block had 19 target trials. In each trial, the stimulus was displayed for 500 ms in the center of screen (resolution: 800 pixels × 600 pixels/inch, refresh rate 60 Hz) with view angle 6.39° × 5.22°. The inter-stimulus interval (ISI) was random between 800 and 1300 ms. A central cross with view angle 2°× 2° appeared in the ISI to help subjects keep fixation of their eyes on the center of screen. Participants were seated comfortably, and requested to minimize eye blinks and body movements during the experiment. To keep attention, they were required to count the number of target stimuli in mind and to report the result at the end of each block. In the experiment, we did not give them the instruction that face perception will be investigated. As the target stimuli randomly appeared among face and non-face stimuli, the subjects have to view all stimuli for identifying the target stimuli. This paradigm is commonly used to eliminate the effect of selective attention on different non-target stimuli (Hillyard et al., 1998). The participants who did not count correctly would be excluded from the analysis.

### EEG Recordings

Scalp EEG were recorded with a NeuroScan v4.3 system using a 62-channel Quik-Cap (NeuroScan, Herndon, VA, USA). Two ocular channels monitored the vertical and horizontal eye movements/blinks from the outer canthi and left infraorbital ridges. Electrode impedances were kept below 5 kΩ. The signals were amplified and digitized at 1 kHz by a SynAmps RT amplifier (Synamps amplifiers, NeuroScan). EEG were first referenced to the common average.

### Data Analysis

#### ERP Analysis

The continuous EEG signals were filtered by a 0.1–50 Hz zero-phase shift band-pass filter using EEGLAB (Delorme and Makeig, 2004). Afterwards, EEG were segmented into 550 ms epochs with 200 ms pre-stimulus baseline plus 350 ms post-stimulus recording. The epochs contaminated by the artifacts (e.g., eye blinks) were removed by using the information from the two ocular channels. The mean baseline value was subtracted from each epoch. ERPs were derived by grand averaging the epochs for each category (faces/buildings/chairs). ERP components, P1, N170, and P2 were measured at occipito-temporal electrodes TP7/8, P7/8, PO7/8, and O1/2. For each subject, the latencies of ERP components were taken at the electrode where the amplitude was maximal over each hemisphere, and the amplitude was measured at each electrode over the ipsilateral hemisphere at that latency. Analysis of variance (ANOVA) with repeated measures was performed to check the statistical significance of ERP results, using stimulus category (faces vs. buildings vs. chairs) and hemisphere (left vs. right) as main factors for both latencies and amplitudes of P1, N170, and P2. Electrode (TP7/8 vs. P7/8 vs. PO7/8 vs. O1/2) was an additional factor only for amplitudes of P1, N170, and P2. Greenhouse-Geisser corrections were made when it is needed in ANOVA. Two-tailed paired sample *t*-tests were used for two-class comparison.

#### Dynamical Functional Connectivity Analysis: Phase Lag Index

The continuous EEG signals were digitally band-pass filtered into four frequency bands, theta (4–7 Hz), alpha (8–14 Hz), beta (15–24 Hz), and gamma (25–45 Hz), with zero-phase shift filters using EEGLAB (Delorme and Makeig, 2004). We computed Hilbert transformed time series of the filtered EEG:

$$x_{H}(t)=\frac{1}{\pi }\text{p}.\text{v}.\int_{-\infty }^{+\infty }\frac{x(\tau )}{t-\tau }d\tau$$

The Hilbert transformed series is related to the original signal by π/2 phase shift with same amplitude and frequency contents. Afterwards, the analytical signal is obtained by:

$$x_{A}(t)=x(t)+jx_{H}(t)=A(t)e^{j\phi (t)}$$

to extract instantaneous phase ϕ(t) of the original signal *x*(t):

$$\phi (t)=\arctan(x_{H}(t)/x(t))$$

We segmented the analytical signal into 300 ms epochs with the period between 30 and 330 ms post-stimulus for PLI analysis. The epochs contaminated by the artifacts were removed by using the information from the two ocular channels as we did for ERP analysis. PLI between all 62-electrode pairs was calculated over epochs at the four frequency bands. PLI is a measure of the asymmetry of distribution of instantaneous phase differences between two signals:

$$PLI=\left|\frac{1}{k}\sum_{k=1}^{k}\text{sign}\left[\Delta \;\phi (t_{k})\right]\right|$$

where Δϕ(t_k_) is instantaneous phase differences between two signals for *k*-epoch, k = 1,....,K, and *K* is the total number of epochs.

Since the zero-lag synchronization was removed by sign function, PLI is less affected by the volume conduction effect compared to traditional functional connectivity measures (e.g., coherence and cross-correlation). The value of PLI ranges between 0 and 1: a zero value means no non-zero phase locking at all, while a PLI value of 1 indicates perfect non-zero phase coupling. Further details on the PLI computation can be found in Stam et al. (2007).

After the computation, PLI values were smoothed over time in 10 non-overlapping 30-ms time windows (30–60 ms, 60–90 ms, …, 300–330 ms) to reduce its fluctuations (Cohen, 2015). We used a 95% confidence threshold (α = 0.05) to determine the significance of PLI. Bonferroni correction was applied to the group analysis. ANOVA was performed to check the significant difference between faces and non-faces, using stimulus category (faces vs. buildings vs. chairs) as the main factor. Greenhouse-Geisser corrections were made when it was needed.

## Results

All subjects reported correct number of target stimuli in the experiment and therefor all subjects were included in the analysis.

### Event-related Potentials (ERP)

**FIGURE 2:**
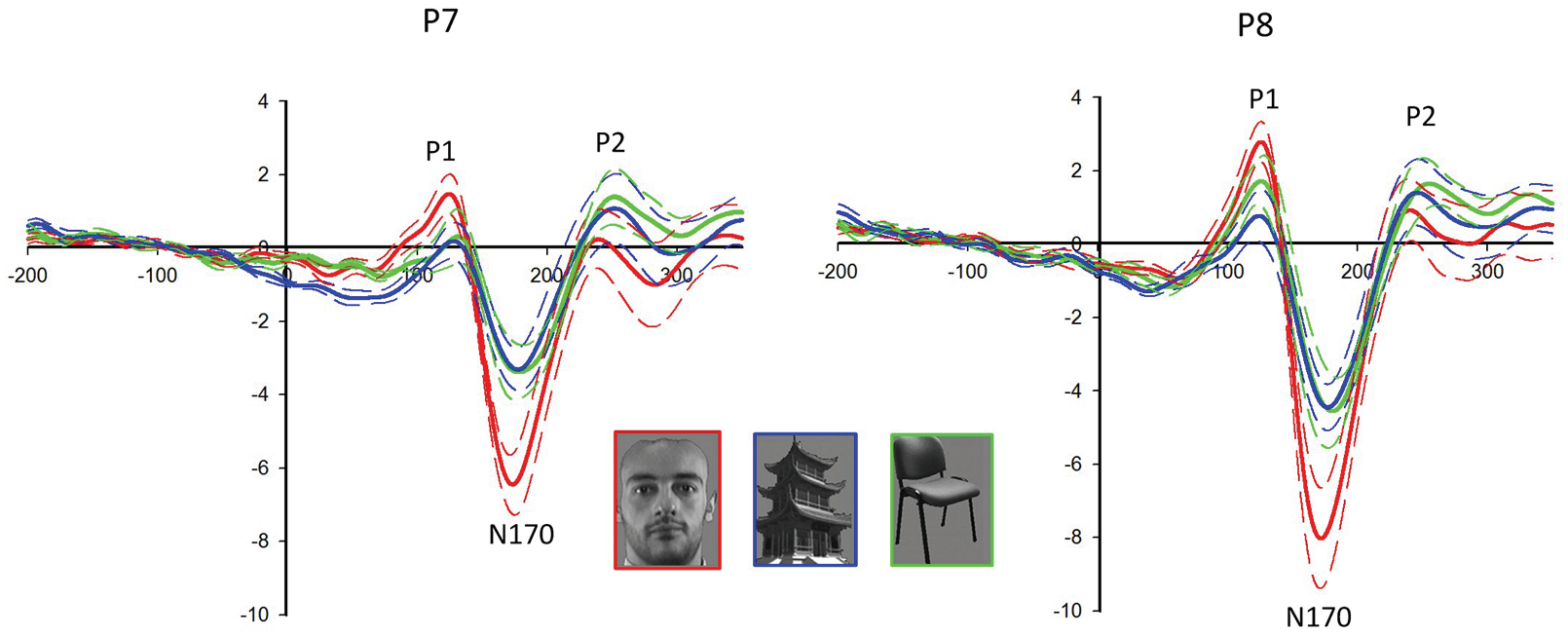
**Event-related potentials (ERPs) at the P7/8 following the presentation of face and non-face stimuli.** The solid lines are the grand averages of 10 subjects. The dashed lines indicate the standard error of the mean. The ERP components P1 and N170 are larger in response to faces (red lines) than non-faces (buildings: blue lines, chairs: green lines) in both hemispheres, with larger amplitudes in the right hemisphere (P8).

Face stimuli elicited the largest P1 (*F*_2,18_ = 25.417, *P* < 0.001) and N170 (*F*_2,18_ = 55.011, *P* < 0.001) among all categories (see **Figure 2**). The latency of N170 was shorter for faces than that for non-faces (*F*_2,18_ = 15.925, *P* < 0.001). Significant electrode effect was shown in the amplitudes of P1 (*F*_1.498,13.478_ = 4.912, *P* = 0.033) and N170 (*F*_1.604,14.432_ = 23.740, *P* < 0.001). For faces, P1 (*F*_1,9_ = 6.589, *P* = 0.030) and N170 (*F*_1,9_ = 7.800, *P* = 0.021) were larger in the right hemisphere than those in the left hemisphere. No significant difference was detected in P2 for the different categories.

### Dynamic Functional Connectivity in Face Perception

**FIGURE 3:**
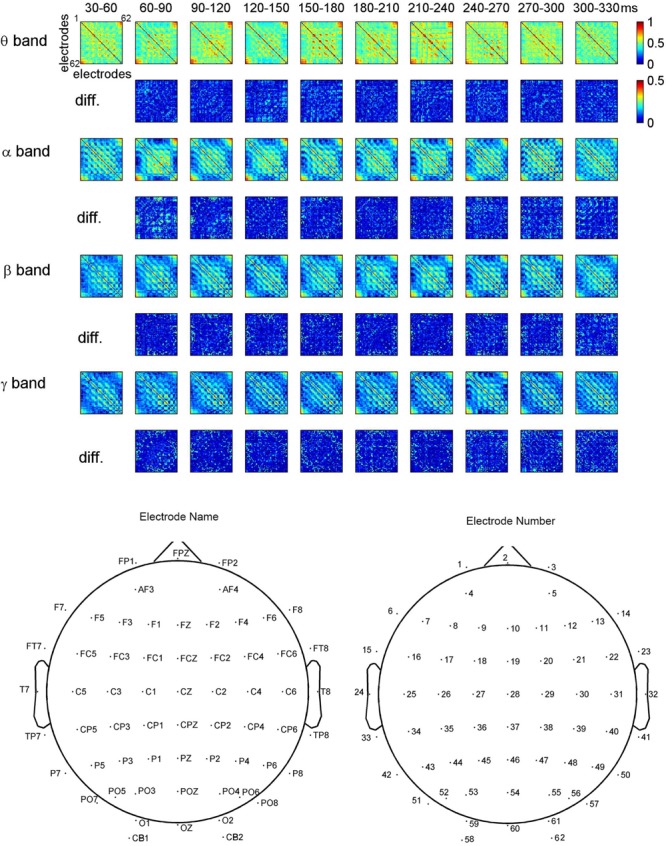
**Dynamic functional connectivity matrices in face perception and the difference between time segments for one representative subject.** The electrodes in the matrices are listed in the ascending order of electrode numbers from top to bottom, and from left to right. For clarity, non-significant phase lag index (PLI) values are set to zero in the maps.

**Figure 3** shows the dynamical functional connectivity between electrodes for different time segments and different frequency bands. The theta band shows stronger functional connectivity than other frequency bands. By computing the difference between neighboring time segments, the dynamic changes of functional connectivity are detected.

**FIGURE 4:**
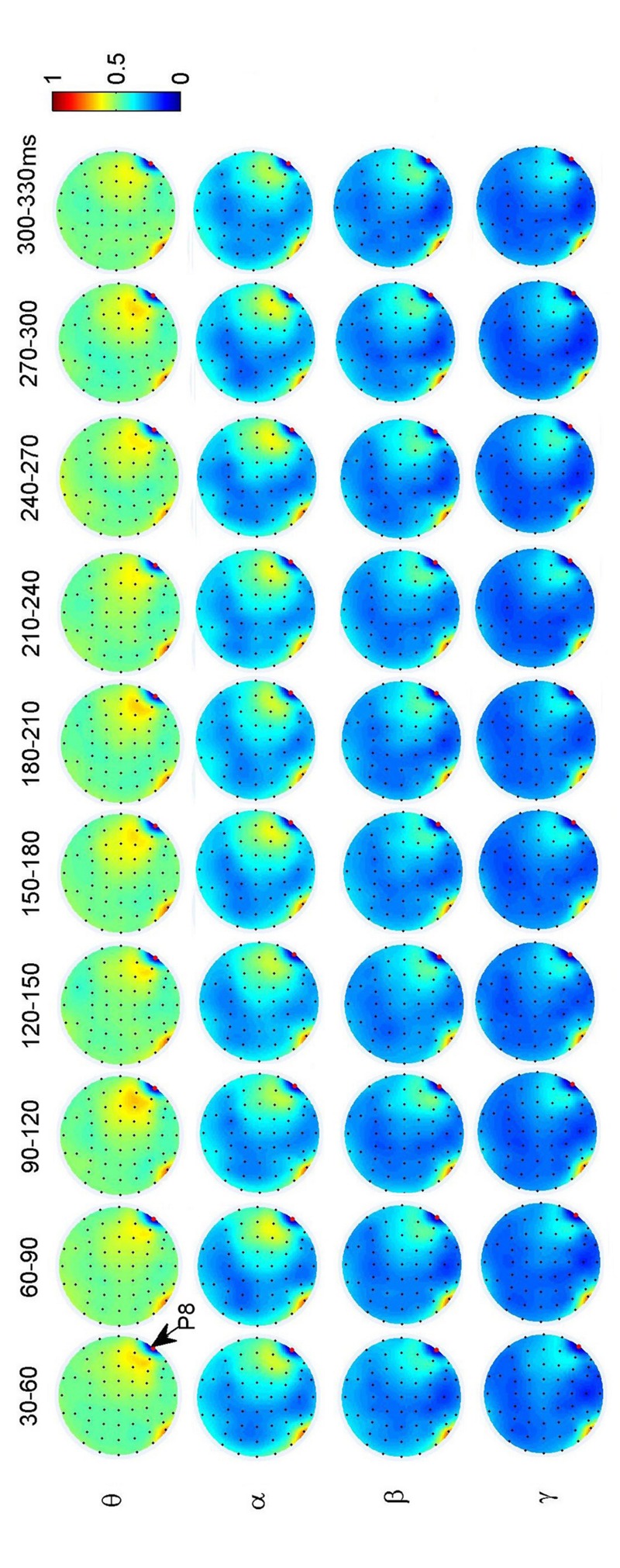
**Grand average of topography of dynamical functional connectivity between the right occipito-temporal electrode P8 (marked out by arrow) and other electrodes over subjects.** Non-significant PLI values are set to zero in the topographies.

**FIGURE 5:**
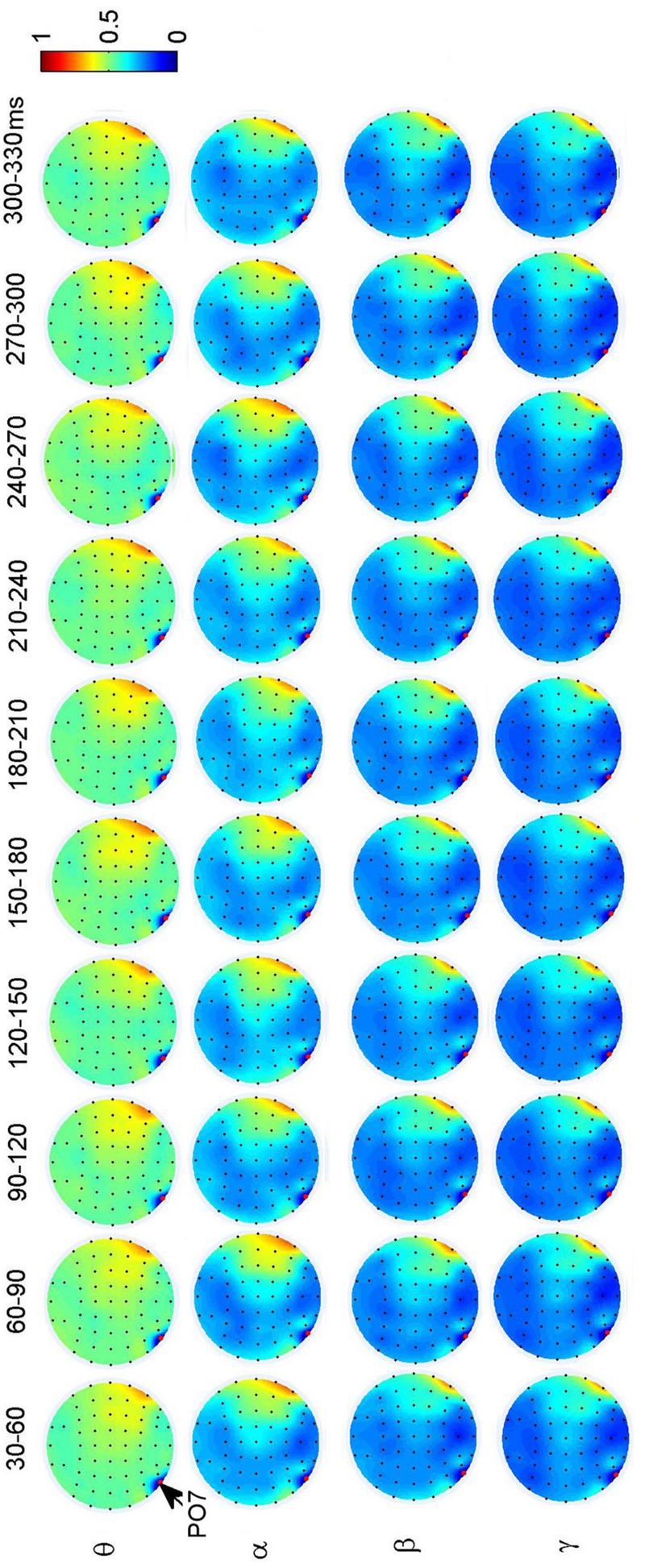
**Grand average of topography of dynamical functional connectivity between the left occipito-temporal electrode PO7 (marked out by arrow) and other electrodes over subjects.** Non-significant PLI values are set to zero in the topographies.

**FIGURE 6:**
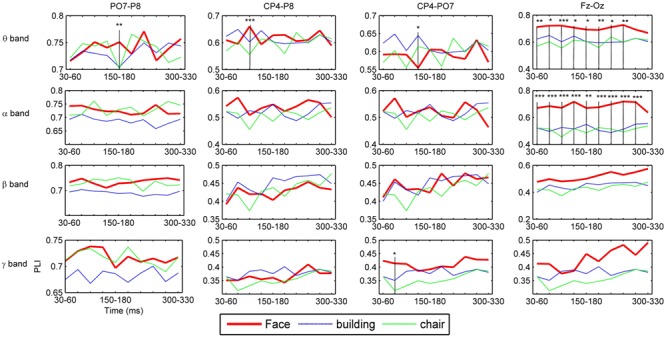
**Grand average of functional connectivity between electrode pairs.** PO7-P8 (between electrodes in bilateral occipito-temporal areas), CP4-P8 (between electrodes in right parietal and occipito-temporal areas), CP4-P7 (between electrodes in right parietal and occipito-temporal areas), and Fz-Oz (between electrodes in prefrontal and occipital areas). The time segment with significant differences between face and non-face stimuli in PLI value are indicated by the vertical lines and stars, where a ‘^∗^’ indicates 0.01 ≤*P* < 0.05, ‘^∗∗^’ 0.001 ≤*P* < 0.01, and ‘^∗∗∗^’*P* < 0.001.

To investigate the dynamic interactions between occipito-temporal areas and other brain areas, the topographies of functional connectivity between right/left occipito-temporal electrodes (P8/PO7) and other electrodes (see **Figures 4** and **5**) are provided for faces in different time segments and frequency bands. We focused on the investigating the dynamic connectivity of face perception. The topography of connectivity was used to serve as a guideline to find the interest areas having significant connectivity with occipito-temporal area, which is known as the ‘core’ system of face perception. The interaction between bilateral occiptio-temporal areas was shown for all time segments and frequency bands. The interaction also exists between right parietal area and bilateral occipito-temporal areas. Further analysis between occipito-temporal electrodes P8 and PO7 indicates the frequency-dependent dynamics, showing different latencies of local peaks for different frequency bands (see **Figure 6**). The local peak values occurred around the ERP latencies of P1 (90–120 ms), N170 (150–180 ms), and P2 (200–230 ms) in the theta band. Statistically significant differences between stimulus classes at the interesting electrode pairs are given in **Figure 6** and indicated by stars. The difference between faces and non-faces was significant in the time segment of 150–180 ms (around the latency of N170) in the theta band (*F*_2,18_ = 6.878, *P* = 0.006), with the largest PLI for faces. The analysis between parietal (CP4) and occipto-temporal area (P8) in the right hemisphere further indicates the interest of theta band (see **Figure 6**). The difference between face and non-face was significant in the time segment of 90–120 ms (around the latency of P1), with the largest PLI for faces (*F*_2,18_ = 11.302, *P* < 0.001). Analysis between the right parietal (CP4) and the left occipto-temporal area (PO7) shows a significant difference between face and non-face stimuli in the time segment of 120–150 ms in the theta band (*F*_2,18_ = 5.299, *P* = 0.016), with lowest PLI for faces (see **Figure 6**). The difference between face and non-face was significant in the gamma band in the time segment of 60–90 ms (*F*_2,18_ = 6.469, *P* = 0.008), with largest PLI for faces.

**FIGURE 7:**
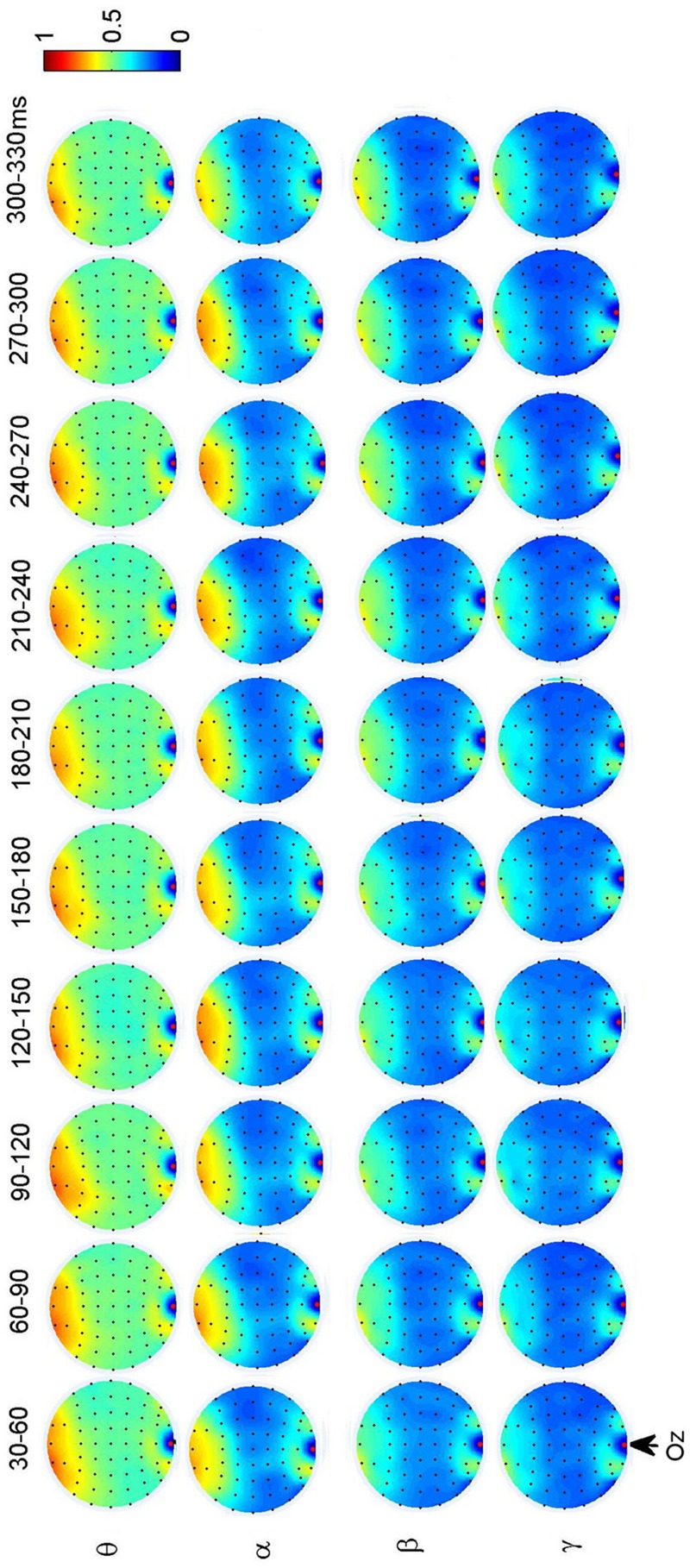
**Grand average of topography of dynamical functional connectivity between the occipito-temporal electrode Oz (marked out by arrow) and other electrodes over subjects.** Non-significant PLI values are set to zero in the topographies.

Additionally, we also found interactions between prefrontal and occipital areas for all frequency bands for faces (see **Figure 7**). The difference between faces and non-faces are significant in wide time ranges in the theta (30–270 ms) and alpha (30–300 ms) bands (see **Figure 6**).

## Discussion

The main focus of this study is to investigate the dynamic functional connectivity between different brain areas during face perception using high-density EEG. To allow for comparison with previous studies we also reported the ERP results in the occipito-temporal electrodes. Face stimuli evoked larger P1 and N170 than non-face stimuli. Although face stimuli evoked ERP responses in both hemispheres, stronger responses were measured in the right hemisphere. These results are in line with previous ERP findings (Bentin et al., 1996; Linkenkaer-Hansen et al., 1998; Latinus and Taylor, 2006; Yang et al., 2011).

Previously, the distributed cortical network for face perception has been revealed using fMRI, showing the static functional connectivity within face-responsive regions, namely the ‘core’ system and between other brain areas, known as the “extended” system (Ishai et al., 2005; Fairhall and Ishai, 2007). Due to the poor temporal resolution of hemodynamic response, the neural dynamics in this network cannot be explored with fMRI. In this study, we used high-density EEG and the PLI to explore the dynamic function connectivity during face perception. EEG is typically very sensitive for volume conduction. However, PLI has been shown to be suitable to assess true functional connectivity from multi-channel EEG, since it is not sensitive to zero phase lag synchronization resulted from the volume conduction effect (Stam et al., 2007). Although a few studies pointed out that zero-lag synchronization can also come from a true but indirect connection (Fischer et al., 2006), e.g., two cortical areas are indirectly connected through the hippocampal relay, this zero-lag connection is thought to be related to cognitive demands and motor acts of lower mammals (Gollo et al., 2011). To best of our knowledge, no such evidence was found in the human brain related to the high-level cognitive process such as face perception. Furthermore, although a few alternative methods, such as phase locking value (Lachaux et al., 1999), can be used to detect zero-lag synchronization (Ioannou et al., 2015), they cannot distinguish the true connectivity from the volume conduction effect (Cohen, 2015). Thus, to best of our knowledge, the advantage of PLI cannot be achieved by using any alternative methods and its potential limitations might not really affect our results.

The changes in the PLI matrices over the time indicate that face perception is mediated by a dynamic network in the human brain. The difference between frequency bands shows the frequency dependence in the network. In general, low frequencies (theta and alpha bands) show stronger phase synchronization than higher frequencies (beta and gamma bands). This is in agreement with previous findings from time-frequency analyses on face perception, where strong EEG spectral modulations and inter-trial phase coherence were detected in the low frequencies (Klopp et al., 1999; Rousselet et al., 2007; Gu et al., 2010).

The connectivity between electrodes in bilateral occipito-temporal areas reflect the inter-hemisphere communication in the “core” system, which has been reported in previous fMRI studies (Minnebusch et al., 2009). The interaction between bilateral occipito-temporal area is continuously shown in the period of 30–330 after stimulus on-set, with local peaks around the latencies of different face processing stages (which is typically reflected by ERP components P1, N170, and P250). Our previous study using independent source analysis and EEG source localization has shown the sequential neural activities of face processing in this period originated from bilateral occipito-temporal area (Yang et al., 2011). Similar evidences can be found in (Deffke et al., 2007a,b). The interactions between the “core” system and the “extended” system are also detected in our study, showing the connectivity between electrodes in parietal and occipito-temporal areas and the connectivity between electrodes in prefrontal and occipital areas.

The topography of functional connectivity between right/left occipito-temporal electrodes and other electrodes showed that only the parietal area in the right hemisphere was involved in the interactions with occipito-temporal areas, which indicate the lateralization of face processing in the human brain. Hemisphere difference in face perception has been reported by both ERP (Bentin et al., 1996; Linkenkaer-Hansen et al., 1998) and functional imaging techniques (Haxby et al., 1995; Fairhall and Ishai, 2007). The right hemisphere is thought to be important in face perception (Ellis and Young, 1983) and may be involved in with ‘deep’ cognitive processing of faces (Hillger and Koenig, 1991; Meng et al., 2012), where the functional role from “extended” system could be highly appreciated.

By comparing the results between faces and non-faces, the significant differences are mainly found in the theta band. The theta oscillations are known to reflect the intrinsic dynamics of thalamo-cortical networks, and are associated with a top-down process for predicting the forthcoming stimulus (Klimesch, 1999; Engel et al., 2001; Osipova et al., 2006; Sauseng et al., 2010). Different from passively viewing an object, face perception is supposed to be mainly driven by a top-down, template-based mechanism instead of a bottom-up, feature analysis (Bentin and Deouell, 2000; Schiltz and Rossion, 2006). This top-down process is likely to be originated in parietal and frontal regions (Mechelli et al., 2004). In this work, we present the evidence from the dynamic function connectivity, where the difference between face and non-face was first shown in the theta band between parietal area and occipito-temporal area in the right hemisphere at the time segment of 90–120 ms, which is around the latency of P1. This difference may link to an initial stage of face categorization (Liu et al., 2002; Herrmann et al., 2005), which occurs before the well-investigated face structural encoding stage (Bruce and Young, 1986). Our previous study indicated that a coarse analysis of face configuration might occur in this initial stage of face categorization (Guo et al., 2013). This coarse analysis simply integrates eyes, nose and mouth into a gestalt, independent of the detailed information of face. After this stage, a decrease of phase synchronization is shown between the right parietal and occipito-temporal areas for faces at the time segment of 120–150 ms, while an increase of phase synchronization between the right parietal and left occipito-temporal areas was present for non-faces. These results indicate that the initial stage of object categorization occurs later than that of face categorization.

We also detected a significant difference in the theta band in the time segment of 150–180 ms (around the latency of N170) for the connectivity between bilateral occipito-temporal areas, showing largest PLI for faces. This timing is known to be the stage of structural encoding (commonly reflected by the N170) of face. Thus, this result suggests an enhanced inter-hemisphere communication in the core system in the stage of face structural encoding, which might be associated with the face-sensitive N170 in the occipito-temporal areas.

**FIGURE 8:**
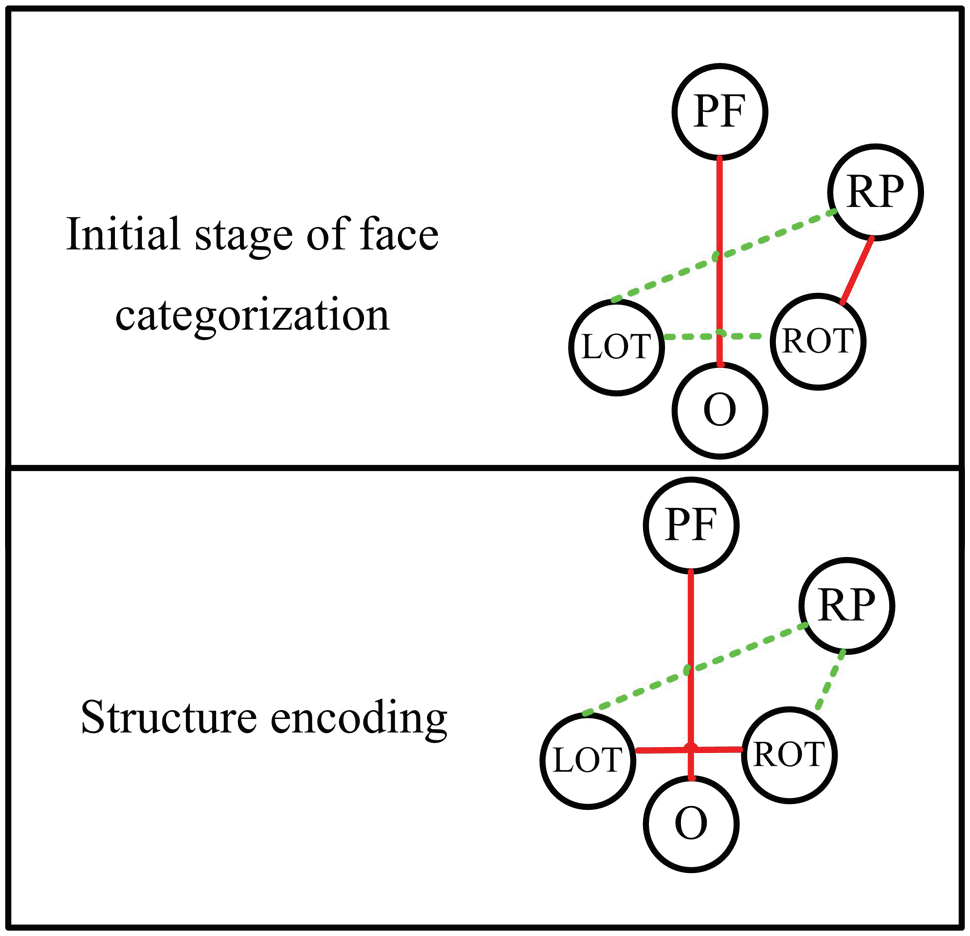
**The dynamic functional network in theta band for different stages of face processing.** PF, prefrontal area; RP, right parietal area; ROT, right occipito-temporal area; LOT, left occipito-temporal area; O, occipital area. Red lines indicate significant stronger connectivity for faces than non-faces, while green lines represent comparable or weaker connectivity for faces.

The significant difference between face and non-face in the theta band connectivity is also shown between prefrontal and occipital area during the whole early face processing period from 30 to 270 ms, showing largest PLI for faces. Since subjects were naïve to the experiment and irrelevant stimuli (butterflies) was introduced to eliminates the effect of selective attention, the difference of connectivity between Fz and Oz is less likely related to task difficulty or selective attention. **Figure 8** summarize a scheme of the dynamic network between different brain areas in the theta band for face-sensitive processing. The face-sensitive prefronto-occipital interaction is also detected in the alpha band. Similar to theta oscillations, the alpha oscillations are also known to be related to top-down mechanism of visual processing (Sauseng et al., 2005, 2006; Jokisch and Jensen, 2007). Both patient and transcranial magnetic stimulation (TMS) studies have provided evidences of activation of prefrontal and occipital areas in face perception (Haxby et al., 1996; Marinkovic et al., 2000; van Honk et al., 2002; Steeves et al., 2006; Pitcher et al., 2007, 2011). Prefronto–occipital interactions may link to a stimulus category-specific top-down modulation during visual perception (Gazzaley et al., 2007), and typically reported in the emotional face perception (Wright et al., 2001; Sergerie et al., 2005; Monk et al., 2008). Our results provide the evidence that the face-sensitive prefronto-occipital interaction also occur in the normal face perception. The continuous strong communication between prefrontal and occipital area in the low frequencies (theta and alpha bands) may greatly facilitate the processing of face information in the human brain.

Besides, the significant difference between face and non-face was shown in the gamma band at time segment of 60–90 ms for the interaction between the right parietal and left occipito-temporal areas. The coherent gamma oscillations are commonly known to associate with the bottom-up, feature-based processing (Busch et al., 2004). Thus, this result possibly reflects the difference between faces and non-faces in the low-level feature processing (likely the context of stimulus, since luminance of stimulus has been uniformed), which typically happens before the initial stage of face categorization (Liu et al., 2002; Guo et al., 2013).

## Conclusion

This study reveals the dynamic functional connectivity between brain areas during passively viewing upright faces. Where previous studies using fMRI focused on a detailed map of the (static) network, our study focuses on the dynamics of brain connectivity using high-density EEG. The results are obtained by using normal faces as stimuli and a model-free approach for brain connectivity. Our results indicate the importance of theta-band connectivity in the face-sensitive processing, which likely links to a top-down, template-based mechanism of face processing. Furthermore our results suggest that the initial stage of face categorization and the stage of face structural encoding are medicated in different parts of the network. This work could be useful to provide a neurophysiological reference for clinic studies related to deficits in face perception.

## Author Contributions

YY conducted the whole study and drafted the manuscript. YQ and AS contributed in the problem identification, data interpretation and editing of the manuscript.

## Conflict of Interest Statement

The authors declare that the research was conducted in the absence of any commercial or financial relationships that could be construed as a potential conflict of interest.
